# Curated Collections for Educators: Five Key Papers about Program Evaluation

**DOI:** 10.7759/cureus.1224

**Published:** 2017-05-04

**Authors:** Brent Thoma, Michael Gottlieb, Megan Boysen-Osborn, Andrew King, Antonia Quinn, Sara Krzyzaniak, Nicolas Pineda, Lalena M Yarris, Teresa Chan

**Affiliations:** 1 Department of Emergency Medicine, College of Medicine, University of Saskatchewan; 2 Department of Emergency Medicine, Rush University Medical Center; 3 Emergency Medicine, University of California at Irvine; 4 Emergency Medicine, The Ohio State University Wexner Medical Center; 5 Department of Emergency Medicine, SUNY Downstate College of Medicine; 6 Department of Emergency Medicine, University of Illinois College of Medicine At Peoria; 7 Department of Emergency Medicine, Universidad San Sebastián; 8 Department of Emergency Medicine, Oregon Health & Science University; 9 Faculty of Health Sciences, Division of Emergency Medicine, McMaster University

**Keywords:** program evaluation, medical education, curated collection

## Abstract

The evaluation of educational programs has become an expected part of medical education. At some point, all medical educators will need to critically evaluate the programs that they deliver. However, the evaluation of educational programs requires a very different skillset than teaching. In this article, we aim to identify and summarize key papers that would be helpful for faculty members interested in exploring program evaluation.

In November of 2016, the 2015-2016 Academic life in emergency medicine (ALiEM) Faculty Incubator program highlighted key papers in a discussion of program evaluation. This list of papers was augmented with suggestions by guest experts and by an open call on Twitter. This resulted in a list of 30 papers on program evaluation. Our authorship group then engaged in a process akin to a Delphi study to build consensus on the most important papers about program evaluation for medical education faculty.

We present our group’s top five most highly rated papers on program evaluation. We also summarize these papers with respect to their relevance to junior medical education faculty members and faculty developers.

Program evaluation is challenging. The described papers will be informative for junior faculty members as they aim to design literature-informed evaluations for their educational programs.

## Introduction and background

Medical educators spend much of their time developing and delivering educational programs. Programs can include didactic lectures, online modules, boot camps, and simulation sessions. Program evaluation is essential to determine the value of the teaching that is provided [1-2], whether or not it meets its intended objectives and how it should be improved or modified in the future [3]. However, rather than beginning at a program's conception [2], evaluation is often only considered late in the process or after the curriculum has been delivered [1].

Program evaluation can be mistaken for assessment or research, but these constructs are subtly different. Within medical education, assessment is generally understood to be the measurement of individual student performance [4]. While student success can provide some information on the effectiveness of a program, program evaluation goes further to determine whether the program worked and how it can be improved [3]. Program evaluation often overlaps and shares methods with research, but its primary goal is to improve or judge the evaluated program, rather than to create and disseminate new knowledge [4].

In 2016, the Faculty Incubator was created by the Academic Life in Emergency Medicine (ALiEM) team to create a virtual community of practice (CoP) [5-6] for early career educators. In this online forum, members of this CoP discussed and debated topics relevant to modern emergency medicine (EM) clinician educators. As part of this program, we created a one-month module focused on program evaluation.

This paper is a narrative review, which highlights the literature that was felt to be the most important for faculty developers and junior educators who wish to learn more about program evaluation.

## Review

### Methods

During November 1-30, 2016, the junior faculty educators and mentors of the ALiEM Faculty Incubator [7] discussed the topic of program evaluation in an online discussion forum. The Faculty Incubator involved 30 junior faculty members and 10 mentors. All junior faculty members were required to participate in the discussion which was facilitated by the mentors, however, participation was not strictly monitored. The titles of papers that were cited, shared, and recommended were compiled into a list.

**Figure 1:**
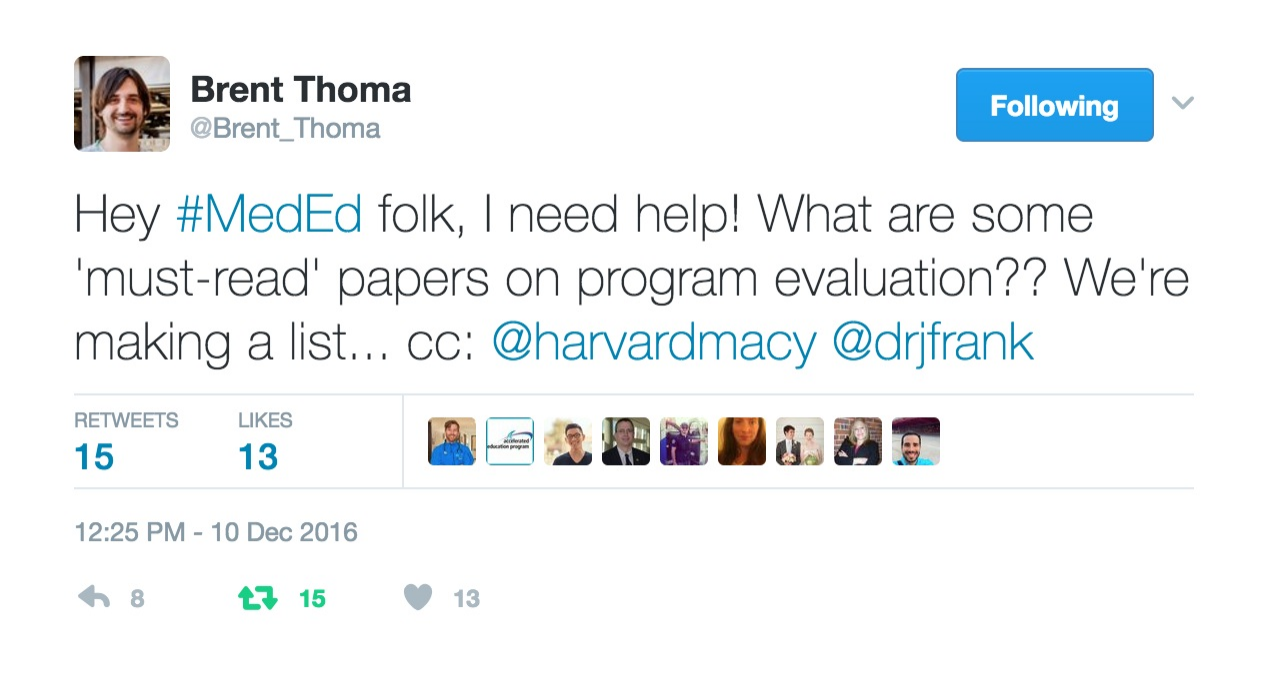
Tweet by Brent Thoma soliciting requests for key papers on program evaluation in medical education

This list was expanded using two other methods: articles recommended during a YouTube Live discussion featuring mentors with significant experience in program evaluation (Dr. Lalena Yarris, George Mejicano, Chad Kessler, and Megan Boysen-Osborn) and a call for important program evaluation papers on Twitter. We ‘tweeted’ requests to have participants of the free open access meducation and medical education (#FOAMed and #MedEd) online virtual communities of practice [8] provide suggestions for important papers on the topic of program evaluation. Figure 1 demonstrates an exemplary tweet. Several papers were suggested via more than one modality.

The importance of these papers for program evaluation was evaluated through a three-round voting process inspired by the Delphi methodology [9-11]. All of this manuscript's authors read the 30 articles and participated in this process. In the first round, raters were asked to indicate the importance of each article on a seven-point Likert scale, anchored at one by the statement "unimportant for junior faculty" and at seven by the statement "essential for junior faculty." In the second round, rates were provided with a frequency histogram displaying how each article had been rated in the first round. They were then asked to indicate if each article "must be included in the top papers" or "should not be included in the top papers." In the third round, rates were provided with the results of the second round as a percentage of raters who indicated that each article must be included. They were then asked to select the five papers which should be included in the article because they are the most important. 

Similar methods were used by the ALiEM faculty incubator in a previous series of papers published in the *Western Journal of Emergency Medicine and Population Health* [12-15]. Readers will note that this was not a traditional Delphi methodology [9] because our rates included novices (i.e. junior faculty members, participants in the faculty incubator) as well as experienced medical educators (i.e. clinician educators, all of whom have published > 10 peer-reviewed publications, who serve as mentors and facilitators of the ALiEM faculty incubator). Rather than only including experts, we intentionally involved junior educators to ensure we selected papers that would be of use to a spectrum of educators throughout their careers.

### Results

The ALiEM faculty incubator discussions, expert recommendations, and social media requests yielded 30 articles. The paper evaluation process resulted in a rank-order listing of these papers in order of perceived relevance as indicated by the results of round three. The top five papers are expanded upon below. The ratings of all 30 papers and their full citations are listed in (Table 1).

**Table 1:** The complete list of study design literature reviewed by the authorship team and the ratings following each round of evaluation

Article Title	Round 1: Mean rating (SD)	Round 2: % of raters that endorsed this paper	Round 3: % of raters that endorsed this paper	Top 5 Papers
Twelve tips for evaluating educational programs [1]	6.8 (0.4)	100%	100%	1st (tie)
Program evaluation models and related theories: AMEE Guide No. 67 [16]	6.7 (0.5)	100%	100%	1st (tie)
AMEE Education Guide no. 29: evaluating educational programmes [4]	6.2 (1.4)	88.9%	100%	1st (tie)
Rethinking program evaluation in health professions education: beyond 'did it work'? [3]	6.0 (1.0)	100%	88.9%	4th
Perspective: Reconsidering the focus on "outcomes research" in medical education: a cautionary note [17]	5.7 (1.2)	77.8%	77.8%	5th
A conceptual model for program evaluation in graduate medical education [18]	5.9 (1.1)	55.6	0%	
Evaluating technology-enhanced learning: A comprehensive framework [19]	5.8 (1.3)	66.7	22.2%	
The structure of program evaluation: an approach for evaluating a course, clerkship, or components of a residency or fellowship training program [20]	5.6 (0.9)	44.4%	0%	
AM last page: A snapshot of three common program evaluation approaches for medical education [21]	5.6 (1.0)	55.6	0%	
Using an outcomes-logic-model approach to evaluating a faculty development program for medical educators [5]	5.0 (1.2)	22.2%	0%	
Achieving desired results and improved outcomes: integrating planning and assessment throughout learning activities [22]	5.0 (1.7)	44.4%	0%	
Diseases of the curriculum [23]	4.9 (1.7)	55.6%	11.1%	
Nimble approaches to curriculum evaluation in graduate medical education [24]	4.7 (1.0)	22.2%	0%	
12 Tips for programmatic assessment [25]	4.7 (2.2)	55.6%	0%	
A model to begin to use clinical outcomes in medical education [26]	4.4 (1.4)	22.2%	0%	
Meta-analysis of faculty's teaching effectiveness: Student evaluation of teaching ratings and student learning are not related [27]	4.4 (1.9)	0%	0%	
Transforming the academic faculty perspective in graduate medical education to better align educational and clinical outcomes [28]	4.3 (1.4)	0%	0%	
How we conduct ongoing programmatic evaluation of our medical education curriculum [29]	4.2 (1.1)	11.1%	0%	
Using a modified nominal group technique as a curriculum evaluation tool [30]	4.1 (1.3)	11.1%	0%	
A new framework for designing programs of assessment [31]	4.1 (1.7)	0%	0%	
Evaluation of a collaborative program on smoking cessation: Translating outcomes framework into practice [32]	3.9 (1.5)	11.1%	0%	
The role of theory-based outcome frameworks in program evaluation: Considering the case of contribution analysis [33]	3.9 (1.8)	0%	0%	
Use of an institutional template for annual program evaluation and improvement: benefits for program participation and performance [34]	3.4 (1.4)	0%	0%	
Instructional effectiveness of college teachers as judged by teachers, current and former students, colleagues, administrators, and external (neutral) observers [35]	3.3 (1.7)	0%	0%	
Student evaluations of teaching (mostly) do not measure teaching effectiveness [36]	3.1 (1.8)	11.1%	0%	
How we use patient encounter data for reflective learning in family medicine training [37]	3.0 (1.0)	0%	0%	
Half a minute: Predicting teacher evaluations from thin slices of nonverbal behavior and physical attractiveness [38]	2.9 (1.6)	0%	0%	
Experimental study design and grant writing in eight steps and 28 questions [39]	2.6 (1.8)	0%	0%	
Early experience of a virtual journal club [40]	2.4 (1.6)	0%	0%	
Cost: The missing outcome in simulation-based medical education research: A systematic review [41]	2.3 (1.0)	0%	0%	

### Discussion

The following is the list of papers that our group has determined to be of interest and relevance to junior faculty members and faculty development officers. The accompanying commentaries are meant to explain the relevance of these papers to junior faculty members and also highlight considerations for senior faculty members when using these works for faculty development workshops or sessions.

*1. The Association for Medical Education in Europe (AMEE) Education Guide no. 29 Evaluating Educational Programmes [4]:* This education guide within medical teacher begins with a brief discussion of the history of program evaluation. It goes on to recommend a framework of evaluation for educators that focuses on the methodology of evaluation, the context of evaluation practice, and the challenge of modifying existing programs with the results the evaluation. This overview includes detailed sets of questions for evaluators to ask about programs that they review. Perhaps the most salient piece of advice from this paper is that improvement even when modesty is valuable.

Relevance to Junior Faculty Member

This is a high-yield read for the junior faculty educator because it provides a succinct and comprehensive overview of program evaluation through the presentation of a framework, which can be adapted by junior faculty educators. Each step within the framework is accompanied by an explanation to assist the reader in understanding the components.

Considerations for Faculty Developers

Faculty developers should be expected to understand program evaluation in the context of its history. This manuscript summarizes the historical program evaluation literature from within and beyond medical education in a way that contextualizes modern controversies and informs current approaches. Faculty developers should use this manuscript to center themselves within the literature. The framework provided may also guide their approach to evaluating the programs of their more junior faculty members.

*2. Program Evaluation Models and Related Theories- AMEE Guide No. 67 [16]:* This guide discusses the three main education theories that underlie various evaluation models (i.e. reductionist theory, system theory, and complexity theory). It begins by describing the purpose of program evaluation, clarifying the definition of program evaluation, and explaining why we evaluate educational programs. The authors conclude that the main purpose of any educational program is change – be it intended or unintended – and defines program evaluation as the “systematic collection and analysis of information related to the design, implementation, and outcomes of a program for the purpose of monitoring and improving the quality and effectiveness of the program.” The guide ends with a description of four evaluation models (i.e. experimental / quasi-experimental models, Kirkpatrick’s four-level model, logic models, and (context/ input/ process/ product model) informed by these education theories.

Relevance to Junior Faculty Members

Change is the most important aspect of any educational program, so measuring change should be the focus of a program evaluation. It is important for junior educators to understand that evaluation should analyze both the intended and unintended change resulting from a program, rather than solely investigating the intended outcomes. By discussing several different evaluation models and their underlying educational theories, this guide will allow the junior faculty educators to choose the best evaluation modality that is most relevant to their individual educational activity.

Considerations for Faculty Developers

This paper may enhance a faculty developer’s foundational knowledge of program evaluation by summarizing its underlying education theories and common models. It may also serve as a frequent reference for faculty developers as they select conceptual frameworks to inform the evaluation of educational programs.

*3. Twelve Tips For Evaluating Educational Programs [1]: *The tips provided in this article can be summarized into three primary themes. Prior to beginning the evaluation, it is important to understand the program, be realistic in what is possible, define the stakeholders, determine the intended outcomes of the program, select an evaluation paradigm, and choose a measurement modality. As evaluation design begins, assemble a group of collaborators who will help to brainstorm, guide the methods used and assist in the piloting of the evaluation. Finally, they recommend avoiding common pitfalls such as confusing program evaluation with learner assessment, evaluating an outcome that is not consistent with the program’s goals, using an unreliable instrument or an instrument without context-specific validity evidence and having unrealistic expectations.

Relevance to Junior Faculty Members

Planning for program evaluation must take place as part of the program design process and not as an afterthought. The 12 tips provide salient advice and a model that is thorough, yet easily achievable for junior faculty educators. While the format of this paper presents only an overview of several complex concepts (e.g. validity evidence), the author provides references for a more in-depth review of these topics.

Considerations for Faculty Developers

Faculty developers will find this concise and clear paper, helpful as both a reference for mentees and to further their own understanding of program evaluation. In addition to foundational tips, the author summarizes advanced concepts that may apply to a faculty developer’s educational practice. Rather than simply presenting a formula for program evaluation, the inclusion of the strengths and weaknesses of various paradigms allows a more nuanced understanding of the gray areas in evaluating educational programs. Referencing the complexities of validity evidence and the potential drawbacks of a patient-related outcome approaches may spark dialogue in faculty development programs and collaborations. Finally, the references included are thoughtful and relevant and would be good additions to faculty developers’ personal libraries.

*4. Rethinking Programme Evaluation in Health Professions Education-Beyond 'Did it Work?' [3]: *This article begins with a provocative analysis of Kirkpatrick hierarchy, establishing the multiple problems that arise when evaluation programs focus solely on outcomes. Beyond the outcome ("Did it work?"), it reinforces the importance of considering the educational theory ("Why will it work?"), the process ("How did it work?"), the context ("What context is the program operating in?”), and unexpected results within the evaluation of a program. In doing so, the authors open the discussion regarding which evaluation approaches might be better suited for different educational programs. More important than finding "the perfect" evaluation model is gaining a holistic view of a program that clarifies the relationship between interventions and their outcomes.

Relevance to Junior Faculty Members

The spirit of this paper is laudable: do not aim to find a single explanation or theory, but familiarize yourself with the literature and determine the best way to evaluate a program within your own context. It will guide junior faculty in their efforts to develop new educational programs within their educational contexts; focusing not only on if a certain program works, but on why it should work, how it worked, and what else occurred. These questions will guide implementation processes and inform future approaches.

Considerations for Faculty Developers

Providing a historical and theoretical overview of program evaluation as a discipline, this article traces the roots of program evaluation. It highlights the importance of going beyond the Kirkpatrick hierarchy to develop a greater understanding of why a program might succeed or fail. The first figure clearly outlines essential elements that explain how theory intersects with implementation and evaluation and is a must read for those who are training program evaluator to their faculty members, to guide them towards richer methods for describing curricula or programs in their scholarly work. Notably, this advice was considered controversial and should be carefully considered [42].

*5. Perspective: Reconsidering the Focus on "Outcomes Research" in Medical Education- a Cautionary Note [17]:* There is an increasing emphasis on higher-level outcomes (e.g. patient outcomes) in educational research which presents challenges to researchers. After discussing the limitations of this approach, the authors offer salient advice for educational research: begin with a study question and proceed in a stepwise fashion to determine the intended outcome and measurement tool, rather than beginning with the measurement tool and working backward. They recommend beginning with Kirkpatrick level one outcomes (e.g. reaction) and sequentially progressing to higher levels (e.g. learning, behavior, and results) [43] throughout a program of research, rather than always striving to find an impact on patient-level outcomes.

Relevance to Junior Faculty Members

There are several challenges and pitfalls associated with developing medical education studies and evaluating patient-level outcomes. While patient-level outcomes will have a role as educational research continues to evolve, they can be difficult to fund without large grants as multi-site involvement is required to obtain adequate power. Lower level outcomes, such as student learning or behavior, remain important for assessments of novel interventions, as well as for isolating the most effective components of an intervention. This is important advice for junior faculty members who are already influenced by the focus on patient-level outcomes within medical research.

Considerations for Faculty Developers

Faculty developers must acknowledge the problems inherent to seeking patient-level outcomes in educational research and program evaluation. Junior faculty members may be inclined to “shoot for the moon” and seek an impact on patient outcomes before first establishing that their program is well received, leads to attitude and behavioral change, and is sustainable.

### Limitations

As with our previous papers [12-15], this study was not designed to be an exhaustive systematic literature review. We attempted to triangulate our naturally emergent list with more papers by utilizing expert consultation and an open social media call, which yielded some important recommended papers. Considering the depth and breadth of our final list, we feel that these adjunctive methods have resulted in an important, if not comprehensive, review of the literature.

## Conclusions

We present five key papers addressing the topic of program evaluation with discussions and applications for junior faculty members and those leading faculty development initiatives. These papers provide a basis from which junior faculty members can design literature-informed program evaluations for their educational projects.
